# Organ-on-a-chip: recent breakthroughs and future prospects

**DOI:** 10.1186/s12938-020-0752-0

**Published:** 2020-02-12

**Authors:** Qirui Wu, Jinfeng Liu, Xiaohong Wang, Lingyan Feng, Jinbo Wu, Xiaoli Zhu, Weijia Wen, Xiuqing Gong

**Affiliations:** 1 Materials Genome Institute, Shanghai University, Shanghai, 200444 China; 2 School of Life Sciences, Shanghai University, Shanghai, 200444 China

**Keywords:** Organ-on-a-chip, Microfluidic chip, Physiological model, Human organs, Stem cell

## Abstract

The organ-on-a-chip (OOAC) is in the list of top 10 emerging technologies and refers to a physiological organ biomimetic system built on a microfluidic chip. Through a combination of cell biology, engineering, and biomaterial technology, the microenvironment of the chip simulates that of the organ in terms of tissue interfaces and mechanical stimulation. This reflects the structural and functional characteristics of human tissue and can predict response to an array of stimuli including drug responses and environmental effects. OOAC has broad applications in precision medicine and biological defense strategies. Here, we introduce the concepts of OOAC and review its application to the construction of physiological models, drug development, and toxicology from the perspective of different organs. We further discuss existing challenges and provide future perspectives for its application.

## Background

Microfluidics is a science and technology that precisely manipulates and processes microscale fluids. It is commonly used to precisely control microfluidic (10^−9^ to 10^−18^ L) fluids using channels that range in size from tens to hundreds of microns and is known as a “lab-on-a-chip” [1–4]. The microchannel is small, but has a large surface area and high mass transfer, favoring its use in microfluidic technology applications including low regent usage, controllable volumes, fast mixing speeds, rapid responses, and precision control of physical and chemical properties [1, 5, 6]. Microfluidics integrate sample preparation, reactions, separation, detection, and basic operating units such as cell culture, sorting and cell lysis [7]. For these reasons, interest in OOAC has intensified [8]. OOAC combines a range of chemical, biological and material science disciplines [9] and was selected as one of the “Top Ten Emerging Technologies” in the World Economic Forum [10].

OOAC is a biomimetic system that can mimic the environment of a physiological organ, with the ability to regulate key parameters including concentration gradients [11], shear force [12], cell patterning [13], tissue-boundaries, [14] and tissue–organ interactions [15]. The major goal of OOAC is to simulate the physiological environment of human organs [16].

Human physiology is the science of studying the functions of the human body and its organ systems. This is of great significance to our understanding of the dysfunction and pathogenesis of the body, and therefore closely aligns with the fields of medicine, drug development and toxicology [17]. The most relevant and direct methods for studying human physiology are in vivo experiments that study human or model organisms. Bodily functions rely on the interaction and adaptation of many lower-level components such as tissues, cells, proteins and genes. It is therefore challenging to reveal the underlying mechanisms of physiological phenomena simply through in vivo studies [18]. In addition, drug development and toxicology require the assessment of the physiological effects of thousands of compounds [19]. Due to the limitations of low-throughput in vivo testing, biologists use in vitro cell culture. Cell culture refers to the growth and maintenance of cells in a controlled environment [20]. For decades, traditional two-dimensional (2D) cell culture systems formed an important platform for life science research. Using 2D systems, the functions of various cells are studied by culturing cells or cell products. However, 2D systems fail to accurately simulate the physiological manifestations of living tissues/organs, intra-organ interactions and microenvironmental factors [21, 22] and often require verification in in vivo animal models. Due to species differences, animal experiments often fail to replicate human experiments [23], and due to both high costs and ethical issues, the use of animals as models for drug testing has come under scrutiny [24]. In preclinical testing, an inadequate description of the human tissue environment may lead to inaccurate predictions of the combined effects of overall tissue function [25]. OOAC was designed to overcome these shortcomings by providing more physiological model systems [26]. OOAC was proposed as a future replacement technology for experimental animal models [27].

This review introduces recent advances from OOAC technology and discusses its future perspectives for cell biological assessments.

### Organs-on-a-chip design concept and key components

#### Design concept

Culture systems require the control of external and internal cell environments [28]. OOAC combined with micromachining and cell biology can control external parameters and accurately simulate physiological environments [16]. Dynamic mechanical stress, fluid shear and concentration gradients are required on the chip. Cell patterning should also be realized to fully reflect physiological processes.

##### Fluid shear force

Microfluidics enables the dynamic culture of cells through micro-pump perfusion, which facilitates the administration of nutrients and timely waste discharge. The dynamic environment in which cells are located is more comparable to in vivo conditions than static culture. In addition, fluid shear stress induces organ polarity [29]. Importantly, OOAC exerts necessary physical pressure on the normal biological functions of endothelial cells [30] by activating cell surface molecules and associated signaling cascades. Similarly, the incorporation of fluid into the OOAC device permits biological assessments at the single organ level [31]. The OOAC system summarizes flow through a simple “rocker” on a chip fluid motion, or through a more complex programmable “pulsatile” format, arranged in a single loop for organization-specific configurations [32].

##### Concentration gradient

At the microscale level, the fluid acts primarily as a laminar flow, resulting in a stable gradient of biochemical molecules, controlled both spatially and temporally. Various biochemical signals driven by concentration gradients exist in biological phenomena, including angiogenesis, invasion, and migration [33–35]. Microfluidics simulate complex physiological processes in the human body by altering flow velocity and channel geometry using microvalves and micro-pumps to achieve stable, three-dimensional (3D) biochemical concentration gradients.

##### Dynamic mechanical stress

Normal day-to-day organ pressure includes blood pressure, lung pressure, and bone pressure. These pressures play a major role in maintaining mechanically stressed tissues such as skeletal muscle, bone, cartilage and blood vessels [36–38]. Microfluidics enable the use of elastic porous membranes to create periodic mechanical stresses. This mechanical stimulation is considered a key determinant of differentiation during physiological processes [39, 40].

##### Cell patterning

The organization of the human body requires a complex and ordered arrangement of multiple cells to form a functional whole body interactions. Microfluidics control cell patterning for the construction of in vitro physiological models with complex geometries. Surface modifications [41], templates [42], and 3D printing [43] contribute to cell patterning on the chip. The 3D printing method enables multi-scale cell patterning by permitting the formation of hydrogel scaffolds with complex channels. The advantage of 3D printing is to allow user-defined digital masks to provide versatility in cell patterns, critical for the in vitro reconstruction of the cellular microenvironment. Li et al. [44] developed methods to achieve rapid heterotypic cell patterning on glass chips using controlled topological manipulations. This method combines a polyvinyl acetate coating, carbon dioxide laser ablation, and continuous cell seeding techniques on a glass chip. This method enables controlled epithelial–mesenchymal interactions. In addition, mesenchymal cells with similar properties can also be patterned on glass chips. This method can be helpful for large-scale investigation and pharmaceutical testing of cutaneous epithelial–mesenchymal interaction and can also be applied to the patterning of other cells.

### Key components

The OOAC involves four key components, including (1) microfluidics; (2) living cell tissues; (3) stimulation or drug delivery; and (4) sensing [45]. The microfluidic component refers to the use of microfluidics to deliver target cells to a pre-designated location and includes a system of culture fluid input and waste liquid discharge during the culture process. Typically, this component is characterized by miniaturization, integration, and automation [7]. The living cell tissue component refers to components that spatially align a particular cell type in the case of 2D or 3D systems. The 3D arrangements are typically created by the addition of biocompatible materials such as hydrogels. These materials can prevent mechanical damage and shape three-dimensional arrangements. [42]. Although the 3D tissue structure more accurately simulates the in vivo situation compared to 2D models, due to the limitations of technology and cost and the assembly of extracellular matrix and the presetting and formation of vasculature, living cell in organ tissues are still mostly cultivated in 2D. For certain tissues, physical or chemical signals are required to simulate the physiological microenvironment, which promotes micro-tissue maturation and function. For example, electrical stimulation can help myocardial tissue maturation [46]. Different signal stimuli can be derived from for drug screening approaches [47]. The sensing component for detecting and compiling data can be an embedded sensing output component or a transparent chip based visual function evaluation system. Peel et al. [48] used automated systems to image multicellular OOACs, producing detailed cell phenotypes and statistical models for measurements. Kane et al. [49] developed a cell system to monitor cells in a 3D microfluidic setting. These assays featured time-lapse imaging microscopy to assess cellular electrical activity through quality control. A meaningful human-on-chip cell model cannot be described and accessed without microsensors-mediated reading of the metabolic state at characteristic points in the system.

### Emerging OOAC technologies

#### Liver OOAC

**Fig. 1:**
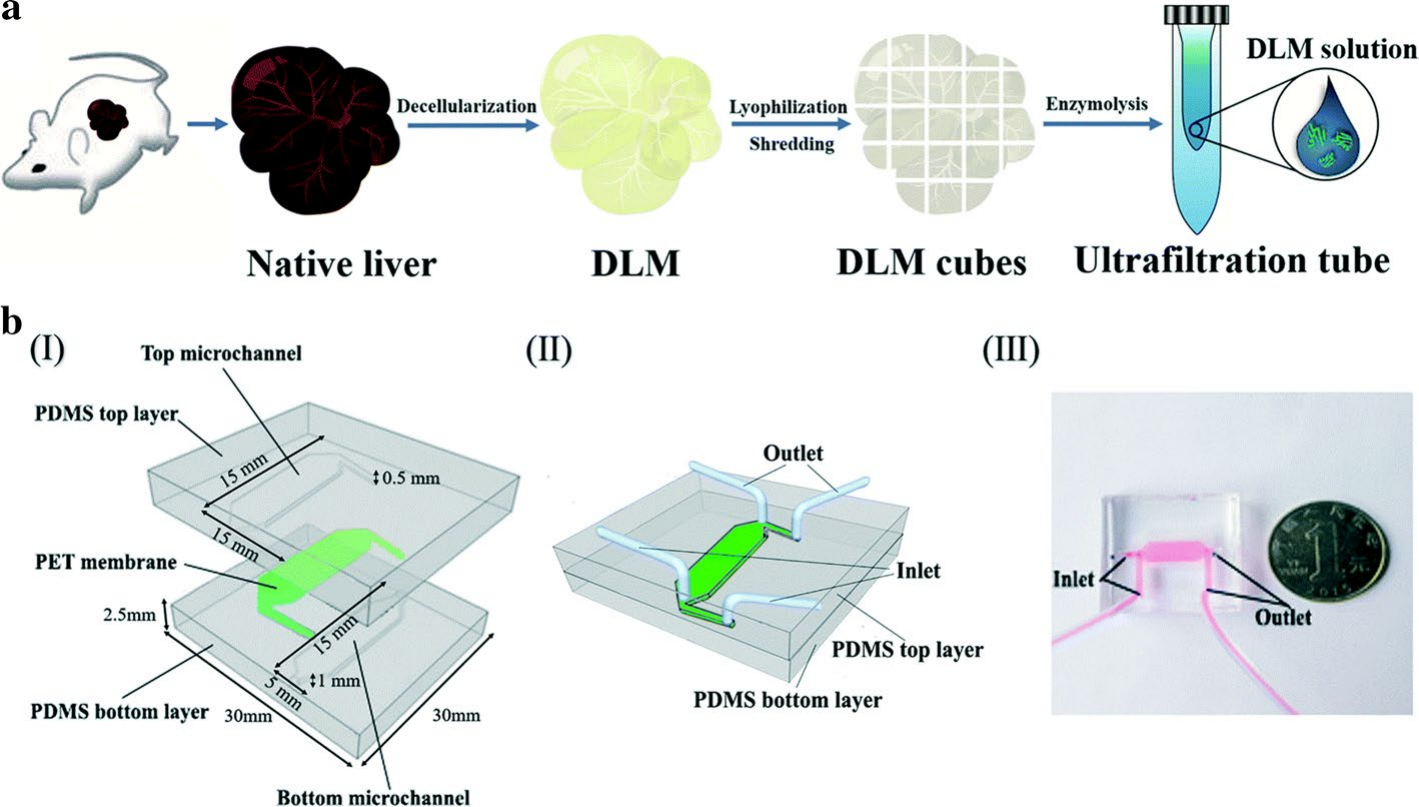
Schematic of the DLM-based liver tumor-on-a-chip. **a** Preparation of the DLM solution from a natural liver; **b** 3D schematic representation of the various components of the equipment (top and bottom, top and bottom microchannels, PET membrane, air inlet, and outlet) and their respective dimensions (reprinted with permission from [52] Copyright © 2006, Royal Society of Chemistry)

The hepatic system is the major site of drug/toxin metabolism. The liver constitutes a series of complex hepatic lobules that confer multicellular functional communication [50]. Maintaining the physiology of hepatocytes over an extended time period is challenging [51]. Kane et al. designed the first liver based system that consisted of microfluidic pores in which 3T3-J2 fibroblasts and rat liver cells were co-cultured to mimic an airway interface (Fig. 1) [52]. Rat hepatocytes cultured in the chip could continuously and stably synthesize albumin and undergo metabolism. Lee et al. [53] designed a chip that mirrored the interstitial structure of endothelial cells and cultured primary hepatocytes, with culture media perfused outside the gap. This permeable endothelial gap separated hepatocytes in cord-based structures permitting their separation from the external sinusoidal region, simultaneously maintaining efficient substance exchange. Ho et al. [13] used radial electric field gradients that were produced using electrophoresis to pattern cells onto circular polydimethylsiloxane (PDMS) chips. These novel techniques simulated the hepatic lobule structure. Hegde et al. [54] fabricated a 2-layer chip that separated the channels using a porous polyethylene terephthalate(PET) membrane and continuously perfused collagen and fibronectin-sanded rat primary hepatocytes into the lower channel through the upper chamber.

To improve the physiological models, 3D hepatocyte culture techniques have been used form microfluidic chips [55]. Ma et al. [56] produced a biomimetic platform for the perfusion of hepatic spheroids in situ. Yum et al. [57] produced systems to study how hepatocytes affect other cell types. High-throughput assays were developed to assess liver cell drug toxicity. Riahi et al. [58] produced microfluidic electrochemical chip immunosensors to detect the biomarkers produced during hepatotoxicity. Chong et al. [59] produced assays to monitor drug skin sensitization through the assessment of metabolite production and the activation of antigen presenting cells (APCs). This system holds value as a drug screening platform to identify compounds that produce systemic skin reactions. Lu et al. [60] developed biomimetic liver tumors through integrating decellularized liver matrixes (DLM) with gelatin methacryloyl (GelMA) to mirror the 3D tumor microenvironment (TME). This system provides an improved disease model for a range of future anti-cancer pharmacological studies. Furthermore, a number of disease or injury states were tested. Kang et al. [61] used their system to analyze viral replication of the hepatitis B virus. Zhou et al. [62] developed a system for modeling alcohol injury. Further characterization of cultured cytoplasm in metabolomics, proteomics, genomics, and epigenomic analysis will help improve the functional outcome of these studies.

#### Lung-on-a-chip

**Fig. 2:**
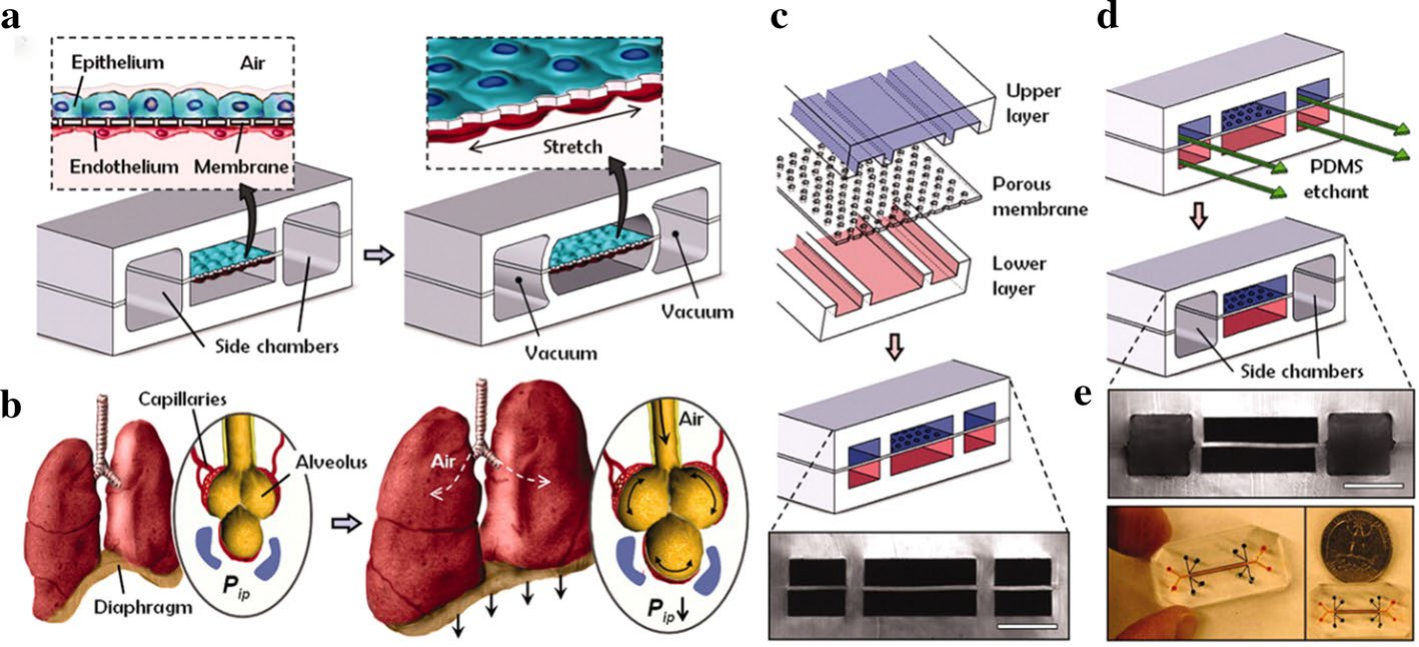
Lung-on-a-chip system. **a** An alveolar–capillary barrier was produced on porous flexible PDMS membranes coated with ECM using spaced PDMS microchannels. The device reproduced respiratory motion through a vacuum leading to mechanical stretching and the formation of an alveolar–capillary barrier; **b** following inhalation, the diaphragm contracts, reducing pleura pressure. The alveolar–capillary interface became stretched due to alveoli tension; **c** device development: a porous membrane between the upper and lower channels bound irreversibly following plasma exposure; **d** PDMS moved through the side of the channels and then was removed following vacuum pressure. **e** Actual images of the device (reprinted with permission from [64] Copyright © 2010, American Association for the Advancement of Science)

Gas exchange in the lungs is regulated by the alveoli which can be challenging to reproduce in vitro. Microfluidics can establish extracorporeal lung models and lung pathologies through accurate fluid flow, and sustained gaseous exchange. Current studies have focused on the regulation of airway mechanical pressure, the blood–blood barrier (BBB), [63] and the effects of shear force on pathophysiological processes. Huh et al. produced a lung-on-a-chip model (Fig. 2) [64] using soft lithography to divide the chip into regions separated by 10 μm PDMS membranes with an extracellular matrix (ECM). The upper PDMS regions had alveolar epithelial cells, whilst the lower regions contained human pulmonary microvascular endothelial cells, thus mimicking the alveolar–capillary barrier. The structures of the membranes were altered under a vacuum to simulate expansion/contraction of the alveoli during respiration. Inflammatory stimuli were introduced into the system through neutrophils that were passed to the fluid channels. This produced a pathological model of pulmonary edema through the introduction of interleukin-2 (IL-2) [65]. This highlights the utility of the OOAC models to improve current in vivo assays.

In 2015, Stucki et al. [66] reported a lung chip that mimicked the lung parenchyma. The system included an alveolar barrier and 3D cyclic strain that mimicked respiration representing the first elastic membrane expansion model to simulate breathing. Blume et al. [67] produced 3D airway culture models that simulated pulmonary interstitial flow through the exchange of both fluid and media. This permitted more in-depth physiological studies of the epithelial barrier. This model utilizes a stent with a permeable filter as a single tissue culture chamber and combined multiple chambers for improved integration. In the lung-on-a-chip, whilst simulating lung gas–liquid interfaces and respiratory dilation through the microfluidic system, pressure can be applied to the alveoli and attached capillaries, providing a shear flow profile. This realistically simulates the lung environment. Humayun et al. [68] cultured airway epithelial and smooth muscle cells at different sides of a hydrogel membrane to assess their suitability as a physiological model. The system was combined with microenvironment cues and toxin exposure as a physiological model of chronic lung disease. Yang et al. [69] produced a poly(lactic-co-glycolic acid) (PLGA) electrospinning nanofiber membrane as a chip matrix for cell scaffolds. Given the ease of the system, it is applicable to lung tumor precision therapy and tissue engineering approaches was highlighted.

Lung tissue organ chips are useful as implantable respiratory assistance devices. Peng et al. [70] designed lung assist devices (LAD) to permit additional gas exchange in the placenta for preterm infants during respiratory failure. The concept of large-diameter channels was achieved in the umbilical arteries and veins, providing LAD with high extra-corporeal blood flow. This has added utility because clinical trials for umbilical vasodilation thresholds were unethical. This study was the first to systematically quantify umbilical vessel damage as the result of expansion by catheters. Dabaghi et al. [71] performed microfabrication for microfluidic blood oxygenators using double-sided gas delivery to improve gas exchange. Oxygen uptake increased to 343% in comparison to single-sided devices. Xu et al. [72] used a microfluidic chip platform to mimic the microenvironment of lung cancer with cancer cell lines and primary cancer cells and tested different chemotherapeutic drugs. Another recent study mimicked asthma in a “small airway-on-a-chip” model [73]. With the models of human asthmatic and chronic obstructive pulmonary disease airways, therapeutics were tested and the chip model recapitulated in vivo responses to a similar therapy.

#### Kidney OOAC

The kidney is responsible for the maintenance of osmotic pressure drug excretion. Kidney toxicity leads to an irreversible loss of renal filtration highlighting the need for drug screening systems. Filtration and reabsorption take place in the nephrons that consist of the glomerulus, renal capsule, and renal tubule. Microfluidics can simulate the fluid environment that support tubular cell growth, and provides porous membrane support for the maintenance of cell polarity [16].

**Fig. 3:**
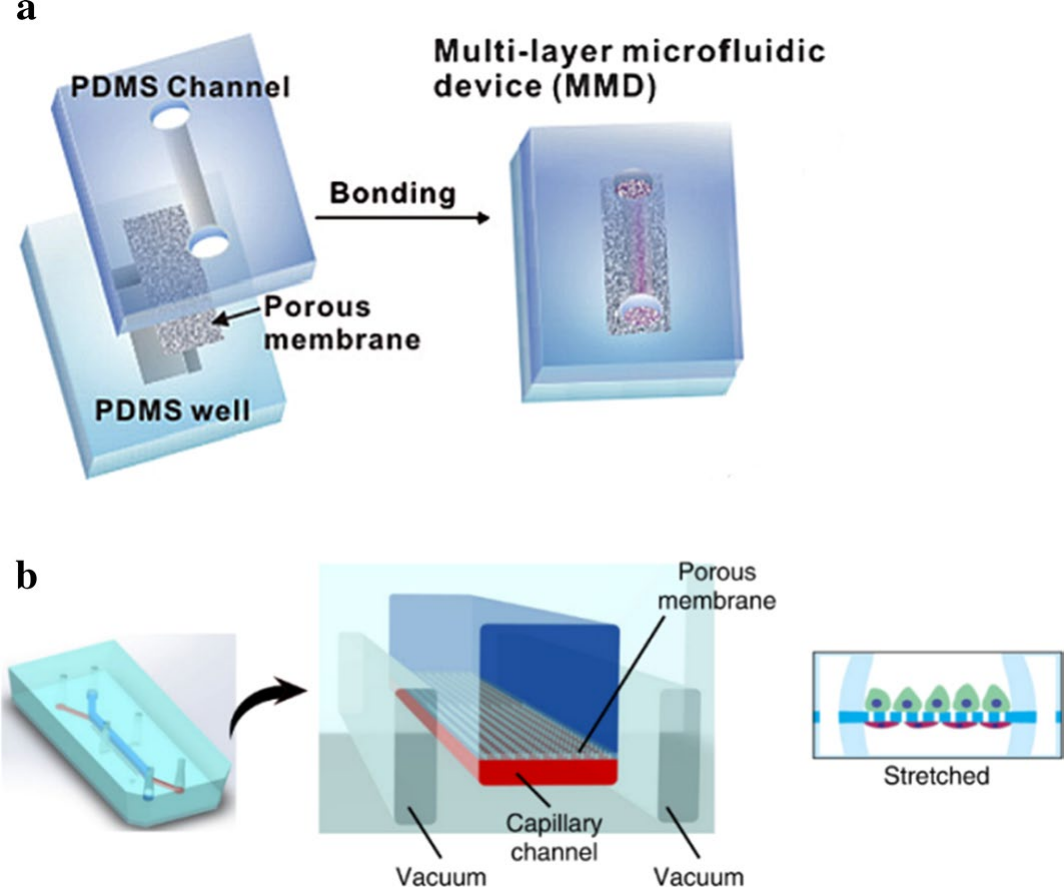
**a** Kidney tubular chip. Sandwich assembly of the PDMS channel, porous membrane, and PDMS reservoir (reproduced from [74]); **b** the channel can replicate the urinary cavity and capillary lumen of the glomerulus. The porous flexible PDMS membrane can be used to functionalize the protein laminin to mimic the glomerular basement membrane. Cyclic mechanical pressure to the cell layer via vacuum stretching of the flexible PDMS film can be produced (reprinted with permission from [76] Copyright © 2018, Royal Society of Chemistry)

Jang et al. [74] produced the first multi-layered microfluidic system (Fig. 3a) in which mouse kidney medullary collecting duct cells were used to simulate renal filtration. The device provided a biomimetic environment that enhanced polarity of the inner medullary collecting duct through promoting cytoskeletal reorganization and molecular transport in response to hormone stimulation. In 2013, the same microfluidic device was used to culture human primary renal epithelial cells [75]. These were the first toxicity studies of primary kidney cells. This device enables direct visualization and quantitative analysis of diverse biological processes of the intact kidney tubule in ways that have not been possible in traditional cell culture or animal models, and it may also prove to be helpful for studying the basic molecular mechanisms of kidney function and disease.

The disadvantage of conventional cell culture systems is that cell differentiation into functional cells requires extended culture times and an external signal detection system. Musah et al. [76] described methods to induce pluripotent stem cell-derived podocytes to form human glomerular chips (Fig. 3b) in organ culture devices. These mimicked the structure and function of the glomerular capillary wall, which was not possible with previously employed methods. The chip was applicable for nephrotoxicity assessments, therapeutic development, regenerative medicine, and kidney development and disease. Sakolish et al. [77] produced a reusable microfluidic chip in human proximal tubules and glomeruli that permitted renal epithelial cells to grow under various conditions. Shear stress causes nephrotoxicity. Schutgens et al. [78] designed stable tubule culture systems that permitted extended expansion and human kidney tissue analysis. Based the system, a multi-purpose primary renal epithelial cell culture model was developed that enabled rapid and individualized molecular and cellular analysis, disease modeling, and drug screening. Tao et al. [79] presented a powerful strategy to generate human islet organoids from human induced pluripotent stem cells. This strategy was applicable to a range of applications for stem cell-based organic engineering and regenerative medicine.

#### Heart-on-a-chip

Cardiovascular deaths are the leading cause of human mortality. The emergence of microfluidics has enabled in vitro bionic studies of cardiac tissue. The myocardium is a major component of the heart. The beating of cardiomyocytes (CMs) can be used to directly assess drug effects and is directly related to heart pumping [80]. In 2012, Grosberg et al. [81] used PDMS to produce an elastic film with a surface texture and implanted neonatal rat CMs on the membrane to form a muscle membranes. As the CMs contract, the muscle film curled to one side. By measuring the degree of this curl it was possible to analyze the differences in the size of the cell contractile capacities on the PDMS film. The experimental system was suitable for both single muscle membrane measurements and high-throughput automated multi-plate assays. Subsequently, in 2013, Zhang et al. [82] utilized hydrogels to produce self-assembled myocardial sheets in a PDMS model. The CMs were derived from differentiated myocardium. Micro-organ tissue chips were produced from 3D printing technology that permitted the integration of myocardial and vascular systems [83]. The model utilized vascular endothelial cells to form vascular networks and CMs were added to the vascular network gap. The organ chip produced a screening platform for CV-related drugs.

**Fig. 4:**
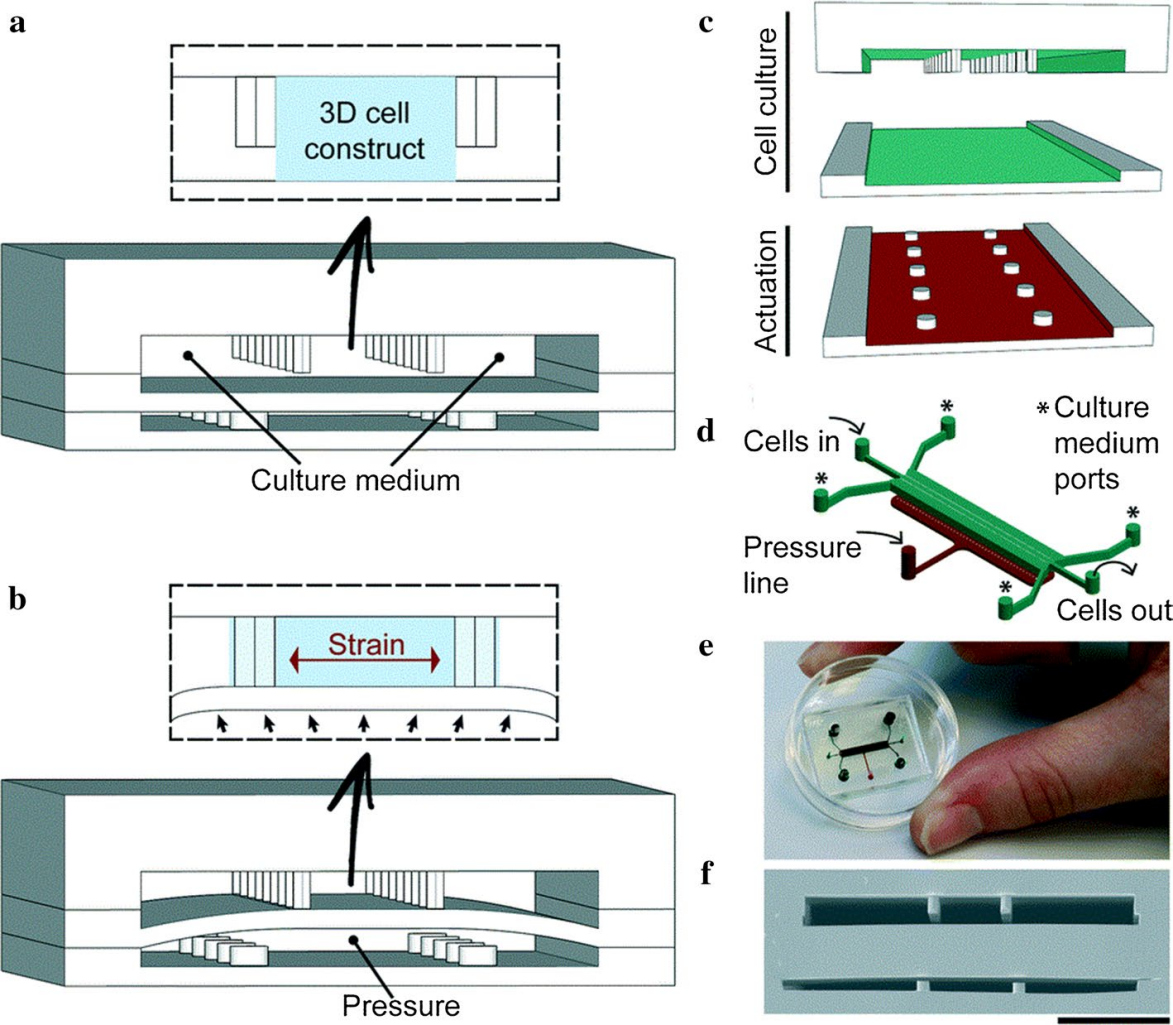
3D heart-on-a-chip. **a** Two separate PDMS microchambers were employed. The CMs are positioned in the central channel to create a 3D construct, whilst the medium is replaced trough side-channels; **b** the lower end of the compartment is pressurized to deform the PDMS membrane and compress the 3D structure. Compression is converted to uniaxial strains applied to the 3D cell structure; **c** PDMS layers are aligned and irreversibly combined. Upper layers are present in the culture chamber and the drive chambers represent the lower layers; **d** 3D illustration; **e** real-life chip; **f** SEM of the chip cross section (reprinted with permission from [85] Copyright © 2016, Royal Society of Chemistry)

Zhang et al. [84] introduced the heart-on-a-chip device that used high-speed impedance detection to assess cardiac drug efficacy. The device records the contraction of CMs to reveal drug effects. The chip represented a preclinical assessment of drug cardiac efficacy. Marsano et al. [85] built a heart organ platform (Fig. 4) that mimicked the physiological and mechanical environment of CMs. Direct visualization and quantitative analysis was performed, which was not permitted in traditional cell culture or animal models. This platform represents an advance in the field and provides standard functional 3D heart models. This makes the device an innovative and low-cost screening platform to improve the predictive power of in vitro models. Schneider [86] designed convenient and efficient chips to generate heart tissue in a controlled environment based on human induced pluripotent stem cells. The viability and function of myocardial tissue was maintained for an extended time period and detailed spatiotemporal pulsation dynamics were optically detected. This platform can be used for a variety of biomedical applications. In addition, Tzatzalos et al. [87] reported that the hiPSC-CMs can represent an unlimited potential for healthy and disease-specific CMs to assess the efficacy of drugs for dilated cardiomyopathy. These advances in drug development have important implications for cardiovascular tissue because cardiotoxicity is often seen in drug trials and is one of the main reasons clinical trials are suspended or drugs are withdrawn from the market.

#### Intestine-on-a-chip

**Fig. 5:**
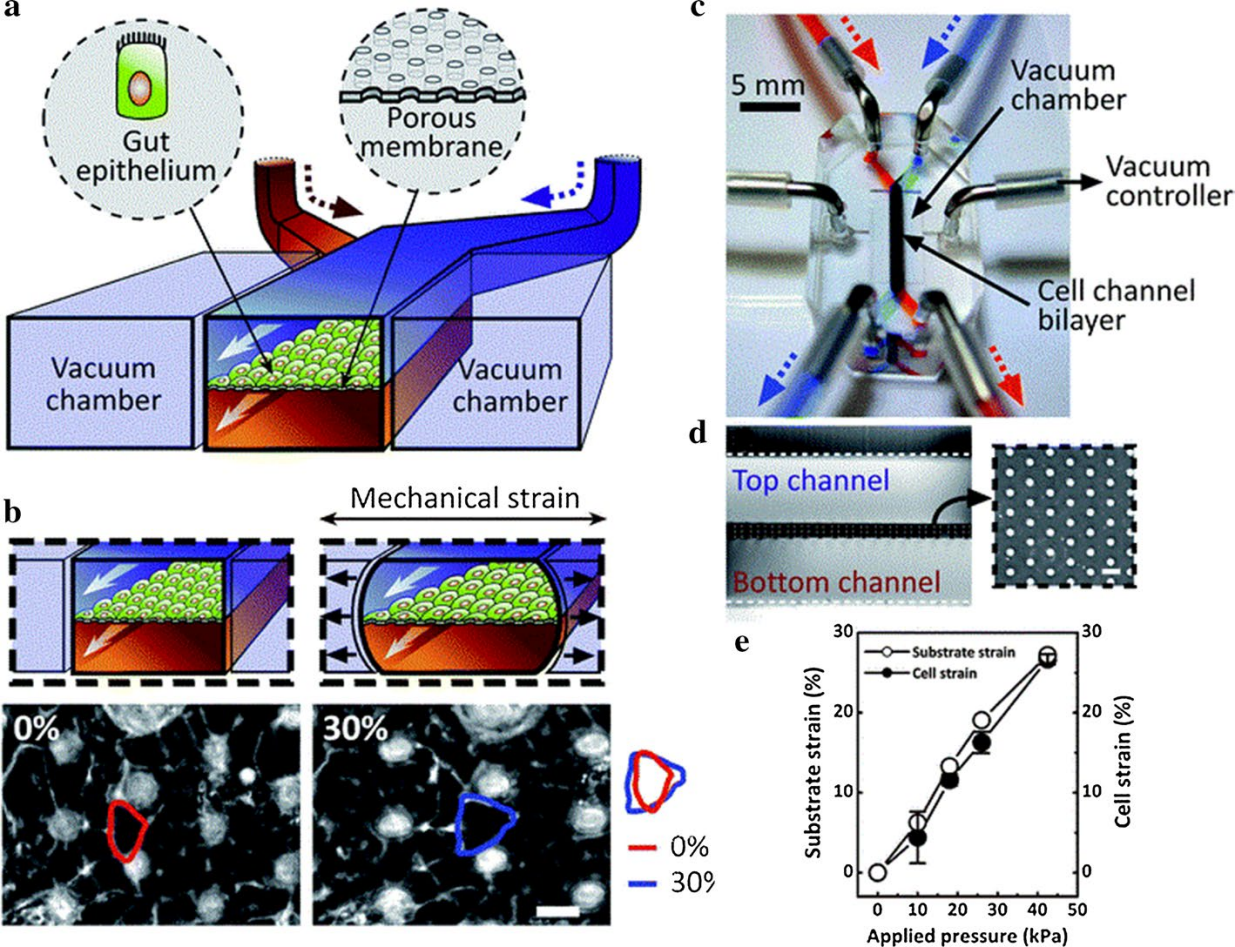
**a** Illustration of the intestine-on-a-chip device; **b** images of the device composed of transparent PDMS elastomers; **c** cross-sectional view of the channels and square illustrations showing a top view of the porous film; **d** schematic of intestinal monolayers cultured on the chips (top) and phase contrast images (bottom) plus (left) or minus (right) mechanical strains (30%); arrows indicate the direction). **e** pressure quantitation (reprinted with permission from [91] Copyright © 2012, Royal Society of Chemistry)

Oral drugs have to transverse the small intestine to enter the bloodstream. Villi are key to absorption and their morphology must be maintained on the chip [88]. Imura et al. [89] developed chips to simulate the intestinal system, consisting of a glass slide permeable membrane and PDMS sheet containing the channels. Caco-2 cells were cultured on the chips. Sung et al. produced the first 3D hydrogel structure to simulate the human intestinal villi [90]. Kim et al. produced bionic devices (Fig. 5) [91]. The microenvironment of the intestine was reconstructed through shear force and cyclic strains. Caco-2 cells show prolonged growth and maintained the microbial flora in the human intestine. The complex structure and physiology of the intestine provided a platform for drug screening and the role of the intestinal microbiome, inflammatory cells and peristaltic-related mechanical deformation during intestinal disease [92]. The device permitted the exploration of the etiology of intestinal disease and identified therapeutic targets and drugs. This study demonstrates the potential of intestine-on-chip for personalized medicine studies on intestinal cells.

Intestinal cells were cultured alone or with endothelial cells including HUVECs [91]. Genome fidelity was low, so the chips mimicked intestinal function. Kasendra1 et al. [93] combined intestinal tissue engineering [94] and OOAC technology to establish in vitro biological models of the human duodenum. The intestinal epithelial cells cultured in the chip were obtained from endoscopic biopsies or organ resections. This chip represented the closest model to the living duodenum and reproduced key features of the small intestine. Recent findings enhanced our knowledge of the intestinal microbiome [95] and intestinal morphology [96].

#### Multi-organs-on-a-chip

An array of physiological pathways requires continuous media circulation and inter-tissue interactions. Single organ chips fail to fully reflect the complexity, functional changes, and integrity of organ function [97]. The “multi-organ-on-a-chip”, otherwise referred to as the “human-on-a-chip” [98] simultaneously constructs multiple organs attracting obvious research attention. Multi-organs-on-a-chip culture cells of different organs and tissues simultaneously which are connected by channels (bionic blood vessel [99]), to achieve multi-organ integration, permitting the examination of interactions to establish a system [100, 101]. These can be separated into static, semi-static and flexible approaches [102]. Static multiple organs are integrated into single connected devices. In semi-static systems, the organs are joined via fluidic networks with Transwell^®^-based [103] tissue inserts. In the flexible system, individual organ-specific platforms are interconnected using flexible microchannels. In such systems, the flexible nature is advantageous and recreates multiple organs [102]. Although the multi-organs-on-a-chip concept remains in its infancy, major breakthroughs have been made, including the design of two-organs [104, 105], three-organs [106, 107], four-organs [108, 109], and ten organs on the chip [110].

**Fig. 6:**
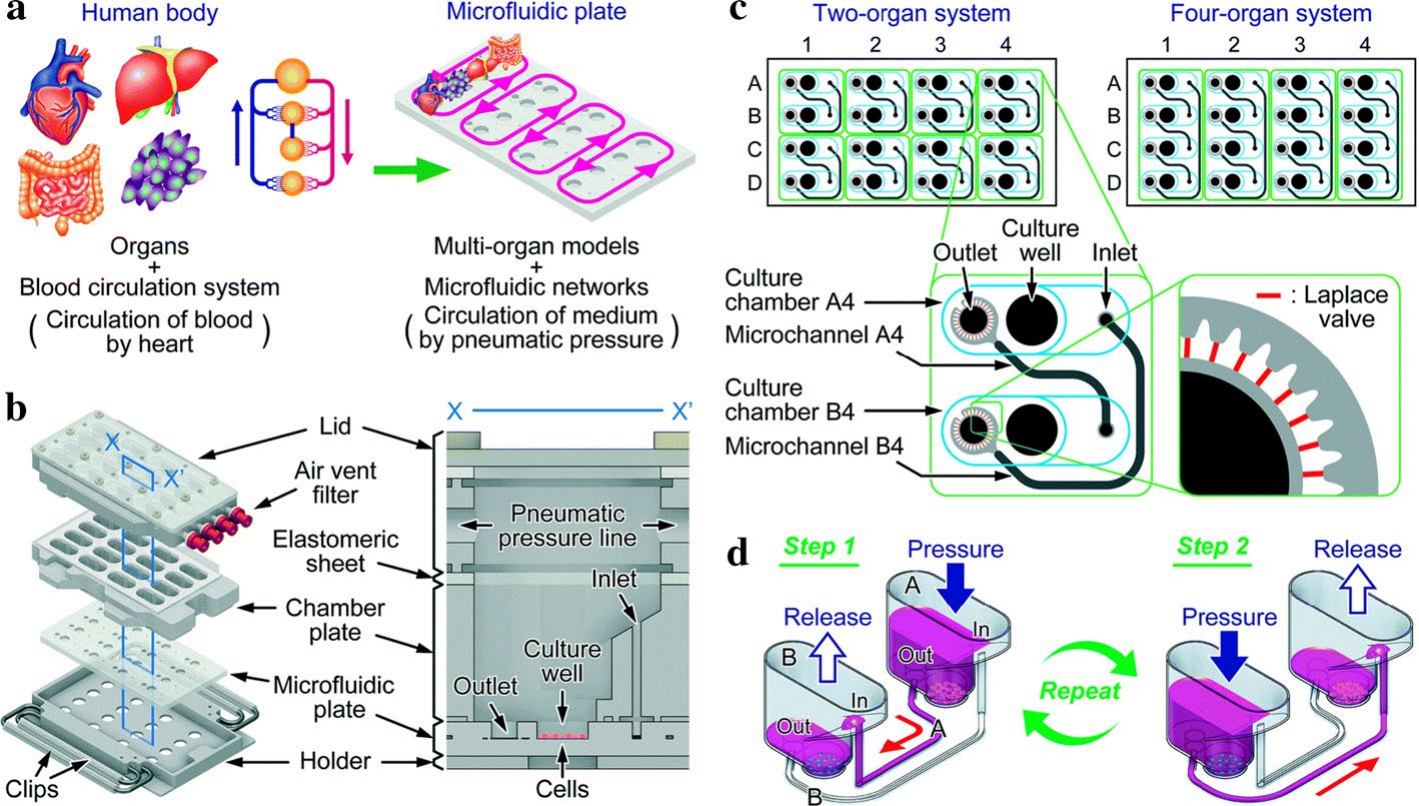
**a** Multi-throughput multi-organ-on-a-plate systems; **b** projection of a culture device containing a 4 × 4 culture chamber illustrated through a culture chamber of an X–X’ cross section; **c** design of microfluidic networks in the microfluidic plates for 8-throughput 2-organ systems and a 4-throughput 4-organ system. Design of the microfluidic networks in microfluidic plates for eight-channel dual-organ systems and four-flux four-organ systems. Closed circles indicate the location of the hole leading to the top surface of the microfluidic plate. Dark and light-shaded areas are deep and shallow microfluidic channels, respectively. Areas surrounded by green lines represent the circulation culture unit. Blue lines indicate the wall of the culture room. Thin red lines surrounding the exit indicate the Laplace valve. **d** Media circulation was performed using pneumatic pressure in the two-organ system. Red arrows indicate the direction of media flow (reprinted with permission from [112] Copyright © 2017, Royal Society of Chemistry)

In 2010, Van et al. [104] were the first to combine liver and intestines in a microfluidic device. The intestine and liver slices functioned on the chip and demonstrated its applicability to organ interactions including the regulation of bile acid synthesis. This system enabled in vitro studies and provided insight into organ–organ interactions. A larger number of organs have since been concentrated onto individual chips. Organ chips are required to maintain stable fluid connection, avoid bacterial contamination, and monitor cell viability throughout the culture process. As the number of organs on the chip increases, the complexity of the system is enhanced, inevitably leading to unpredictable results. Simplifying existing systems is critical to achieving a wider range of applications. Lee et al. [111] fabricated pumpless, user-friendly multi-organs-on-a-chip which were easily assembled and operated. Satoh et al. reported a multi-throughput multi-organ-on-a-chip system formed on a pneumatic pressure-driven medium circulation platform that was microplate-sized (Fig. 6) [112]. This system possesses the following advantages for application to drug discovery: simultaneous operation of multiple multi-organ culture units, design flexibility of the microfluidic network, a pipette-friendly liquid handling interface, and applicability to experimental protocols and analytical methods widely used in microplates. This multi-organ culture platform will be an advantageous research tool for drug discovery.

The continued development of OOAC was dependent on advances in design, modeling, manufacturability, and usability. Lantada et al. [113] produced an innovative combination of laser technologies. The assessment of human mesenchymal stem cells verified the effectiveness of the technique and the resultant chip was transparent, facilitating imaging procedures. Such technologies are feasible for mass-produced chips and hold utility for energy, transportation and aerospace industries.

OOAC technology has developed rapidly in recent years and has enhanced our knowledge of all the major organs. Others not discussed in this review include blood vessels [99, 114, 115], the skin [116, 117], the BBB [118, 119], skeletal muscle [120, 121], and the CNS [122, 123].

### Stem cell engineering

**Fig. 7:**
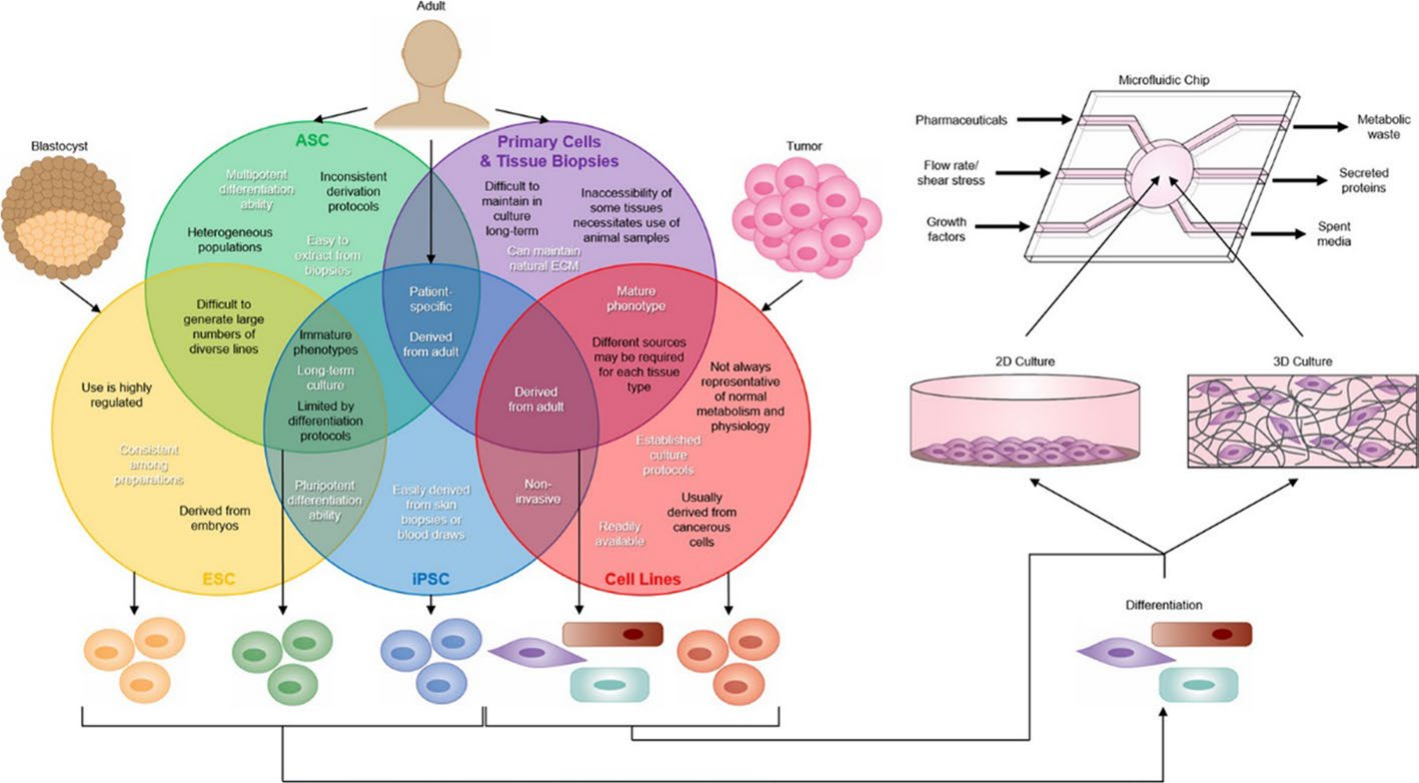
Tissue sources for the organ-on-a-chip (OOAC) devices. Embryonic stem cells (ESCs), induced pluripotent stem cells (iPSCs), and adult stem cells (ASCs) can be differentiated and integrated into microfluidic chips as for cell lines and primary cells. The figure illustrates the advantages (white) and limitations (black) of ESCs, ASCs, iPSCs, primordial and tissue biopsies, and cell lines in OOC devices. Cell lines and primary cells are more common in oocytes as they typically display good biological response characteristics. However, cell lines do not represent normal physiological conditions and primary cell culture time is limited, and the quality is unstable. In contrast, stem cells are readily available and are an infinite cell source. Even with current limitations on differentiation and maturation protocols, stem cells represent a promising technology that can be incorporated into OOC devices (reprinted with permission from [125] Copyright © 2019, Elsevier)

The source of biological tissue is one of the most important parameters in OOAC design. Stem cells can be extracted from humans without tissue biopsy [124]. By definition, a stem cell is any cell that is self-renewing and has the potential to differentiate into one or more specialized cell types. The most common types include embryonic stem cells (ESCs), induced pluripotent stem cells (iPSCs), and adult stem cells (ASCs). These cells can be used as a biological tissue source for OOAC (Fig. 7) [125]. The most common human ASCs are mesenchymal stem cells (MSCs) which are pluripotent stem cells extracted from adult tissue [126]. Bone marrow mesenchymal stem (bMSCs) cells are typically derived from bone marrow or adipose tissue, making them an attractive option due to their ease of extraction from tissue biopsies [127]. Due to their limited ability to differentiate, lack of consistent derivation protocols and clear biological responses, MSCs are less useful in OOAC models than their pluripotent counterparts. Human ESCs originate from blastocysts or internal cells of the embryo. Dependent on the source, they can be pluripotent and differentiate into any type of adult cell from any of the three germ layers [128]. However, human ESCs must be derived from human embryos which is ethically controversial, in turn leading to regulations and restrictions. Due to the ethical debate surrounding ESCs and the technical difficulties of producing large numbers of genetically diverse cell lines, it is more difficult to apply human ESCs to clinical trials than their use as precision drug replacements in disease models for therapeutic drug evaluation [129]. Like ESCs, MSCs are pluripotent and can differentiate from all three germ layers [130]. As iPSCs are derived from adult tissue rather than embryonic tissue, they avoid the ethical issues associated with ESCs. No significant differences in gene expression levels, surface marker expression, and morphology between ESCs and iPSCs are observed in cells from the same genetic background [131, 132]. In addition to circumventing ethical controversies, another advantage of iPSCs over ESCs is that they can be obtained from donors of known disease phenotypes, which can be used for patient-specific disease models and drug screening.

**Fig. 8:**
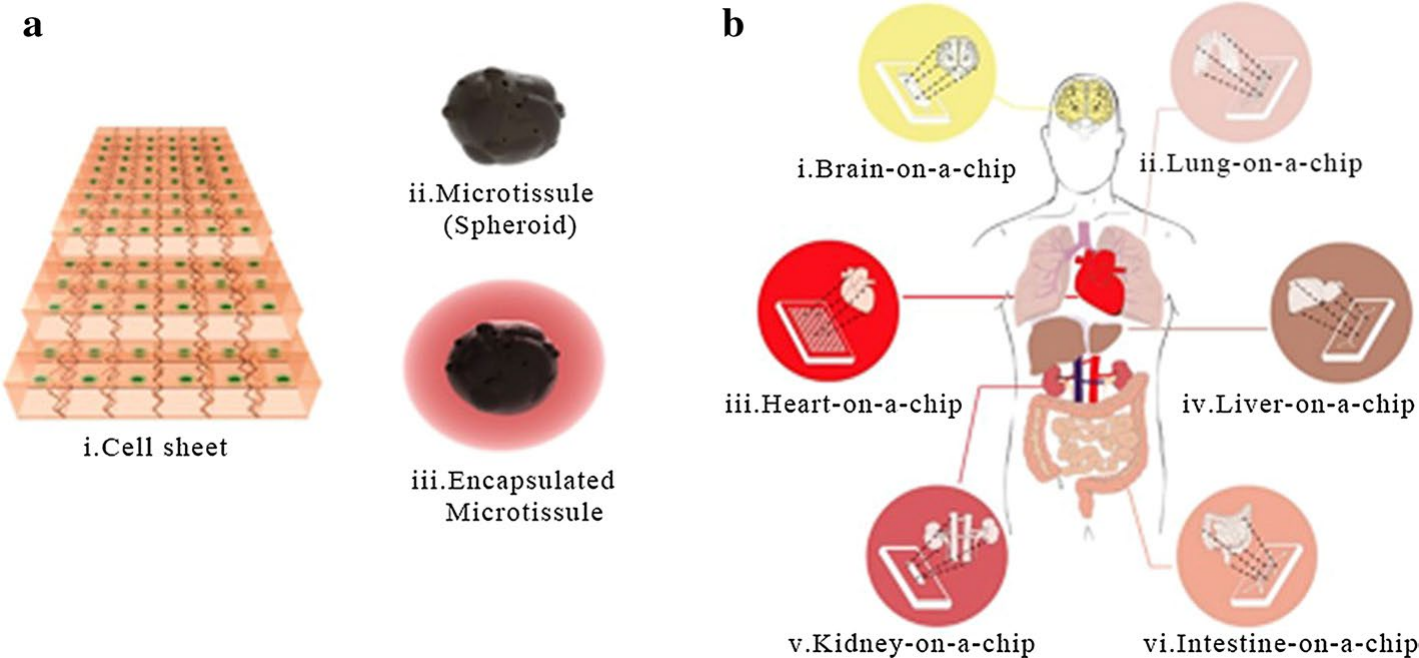
Future trends in stem cell research. **a** Building blocks. **b** Organ-on-a-chip techniques can mimic real-life in vivo states (reprinted with permission from [133] Copyright © 2015, John Wiley and Sons)

Since stem cells are more readily available than many primitive cell types and tissue biopsies, and they are more physiologically representative than other cell lines and are likely to become the main tissue source for future OOAC (Fig. 8) [133]. Continued research into the methods by which stem cells differentiate into functional organ models on chips will contribute to improvements in stem cell methods and advances in OOAC technology [125, 134].

## Conclusion and future perspectives

We have reviewed recent progress in OOAC technology. Microfluidic chips provide favorable support for the development of OOAC. Its development has attracted worldwide research attention and great scientific advances have been made. A large number of OOACs have been designed and prepared. An array of human organs has been studied. The ultimate goal of OOAC is to integrate numerous organs into a single chip, and to build a more complex multi-organ chip model, finally achieving a “Human-on-a-chip”.

Although OOAC technology has developed rapidly, the human-on-a-chip theory remains distant. PDMS is the most widely employed material, but comes with disadvantages as the resultant film is thicker than the in vivo morphology. A decreased absorbance of small hydrophobic molecules influences solvent efficacy and toxicity. It is thus necessary to identify suitable alternative materials. At present, the cost of manufacturing and experimental implementation is relatively expensive, which is not conducive to the widespread use of organ chips, so components must be of low cost and easy to dispose. More expensive components should be reusable. In terms of integrated system components, the media volume and connector size must be reduced for general use. Collecting samples on the chip may interfere with its operation, resulting in changes in the concentration of various metabolites. More suitable sensors are thus required. Universal cell culture mediums suitable for all organs are also required. Most critically, as the number of organs on the chip increases, functionality becomes more complex and generated data carry artefactual and non-translatable risks. This is currently unsolvable. In the case of long-term repeated administration or on-chip studies, the biomarkers identified in vitro may not fully reflect the in vivo equivalent.

## Data Availability

Not applicable.

## Abbreviations

OOAC: Organ-on-a-chip
2D: Two-dimensional
3D: Three-dimensional
PDMS: Polydimethylsiloxane
PET: Polyethylene terephthalate
APCs: Antigen presenting cells
DLM: Decellularized liver matrixes
GelMA: Gelatin methacryloyl
TME: Tumor microenvironment
BBB: Blood-blood barrier
ECM: Extracellular matrix
IL-2: Interleukin-2
PLGA: Poly(lactic-co-glycolic acid)
LAD: Lung assist devices
CMs: Cardiomyocytes
ESCs: Embryonic stem cells
IPSCs: Induced pluripotent stem cells
ASCs: Adult stem cells
BMSCs: Bone marrow mesenchymal stem
